# Case report: Parsonage-turner syndrome in a melanoma patient treated by BRAF/MEK inhibitors after immune checkpoint inhibitors

**DOI:** 10.3389/fonc.2023.1268693

**Published:** 2023-12-19

**Authors:** Charlotte Bonnefin, Fanny Duval, Marie Rouanet, Marie Kostine, Emilie Gerard

**Affiliations:** ^1^ Department of Dermatology, Centre Hospitalier Universitaire (CHU) de Bordeaux, Bordeaux, France; ^2^ Atlantique Occitanie Caraïbe (AOC) Referral Center for Neuromuscular Diseases, Neurology and Neuromuscular Diseases Department, Filière Neuromusculaire (FILNEMUS), Bordeaux University Hospital, Bordeaux, France; ^3^ Department of Neurology, Centre Hospitalier Universitaire (CHU) de Bordeaux, Bordeaux, France; ^4^ ImmunoConcEpT, Université (Univ.) Bordeaux, Bordeaux, France; ^5^ Department of Rheumatology, Bordeaux University Hospital, Bordeaux, France

**Keywords:** neurological adverse event, BRAF/MEK inhibitor, melanoma, encorafenib plus binimetinib, brachial plexus neuritis, parsonage-turner syndrome

## Abstract

**Introduction:**

Combination molecular BRAF/MEK inhibitors targeted therapy has been shown to improve overall survival in patients with BRAF V600 mutated unresectable or metastatic melanoma. Most patients treated with BRAF/MEK inhibitors will experience adverse events but neurological adverse events (nAEs) remain rare.

**Case report:**

A 42-year-old woman diagnosed with metastatic melanoma presented with an intense pain in the left shoulder 7 days after the beginning of encorafenib/binimetinib after immune checkpoint inhibitors (ICI) combination. No other triggering factors were identified. Electromyogram performed one month after the pain onset revealed a left brachial plexopathy suggestive of a Parsonage-Turner syndrome. The weakness slowly improved with intensive rehabilitation and targeted therapies were continued.

**Conclusion:**

We report the first case of Parsonage-Turner syndrome in a melanoma patient treated with encorafenib/binimetinib following checkpoint inhibitors combination.

We cannot rule out the implication of ICI in the development of this syndrome but the rapid onset of the symptoms after the beginning of targeted therapies makes their involvment more likely.

Given the increased use of BRAF/MEK inhibitors in managing of stage III and IV melanoma, as well as the development in stage II, clinicians should be aware of this potential side effect.

## Introduction

Roughly half of advanced cutaneous melanomas harbor a V600 mutation in the BRAF gene, which activates the mitogen-activated protein kinase (MAPK) pathway. Targeted mutation-based therapy with BRAF/MEK inhibitors is among the systemic treatment options for advanced metastatic melanoma, as well as for adjuvant therapy in stage III melanoma following complete resection (1, 2). Common adverse events comprise fever, fatigue, joint pain, rash, loss of appetite, nausea, and diarrhea. Neurological adverse events (nAEs) related to BRAF/MEK inhibitors have frequently been reported, including Guillain-Barre syndrome (GBS), peripheral neuropathies, and facial palsy. Nevertheless, these toxicities remain relatively rare. Since most patients do not achieve long-lasting benefits from BRAF/MEK inhibitors, immune checkpoint inhibitors (ICIs) are usually used as the primary first-line treatment. In patients who have undergone sequential therapies with ICIs followed by BRAF/MEK inhibitors, diagnosing and establishing causality for neurological adverse events can be challenging. We present the first documented case of Parsonage-Turner syndrome in a melanoma patient who received encorafenib/binimetinib following combination checkpoint inhibitors.

## Case report

**Figure 1:**
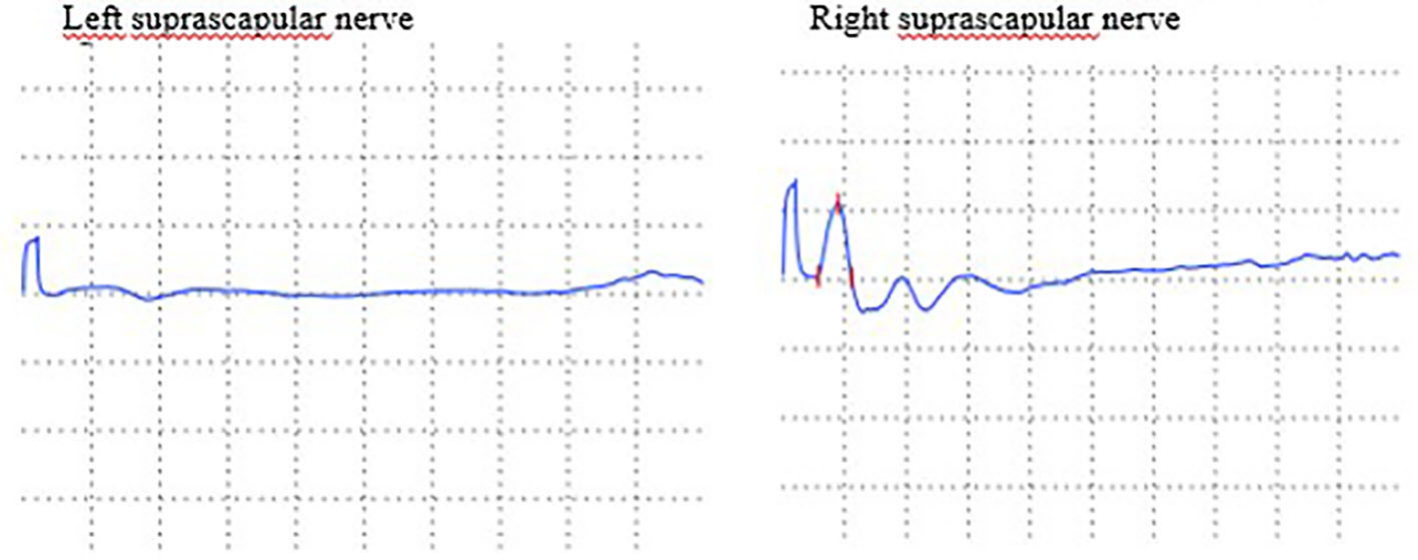
Nerve conduction velocity of motor branch of suprascapular nerves: decrease of amplitude of left suprascapular nerve.

A 42-year-old woman had a medical history of melanoma excised in 2002. Following the surgery, she was placed on active surveillance. In 2021, she developed a left inguinal lymph node and liver nodules, which were confirmed to be metastatic melanoma positive for the BRAF V600E mutation through biopsy. Initially, she received a combination of immune checkpoint inhibitors (ICI), specifically anti-PD-1 and anti-CTLA-4, for nearly three months, with the only side effect being hypothyroidism. Due to disease progression under ICI treatment, she was switched to encorafenib/binimetinib (E/B). On the fourth day of E/B treatment, she developed serous retinopathy, which resolved after a brief temporary discontinuation of the targeted therapy. This allowed the resumption of the E/B regimen at a reduced dose. One week later, she experienced severe acute pain in her left shoulder, scoring 8 on the Visual Analog Scale, without any traumatic context. During the medical examination, she displayed no motor deficits, no movement limitations, and her deep tendon reflexes remained intact. Both X-ray and ultrasound examinations of the shoulder were normal, but a C5-C6 discopathy was noted on cervical spine X-ray. The patient received treatment with analgesics and non-steroidal anti-inflammatory drugs (NSAIDs), which led to the resolution of pain within one month. However, this was followed by muscle weakness, prompting further investigations. An MRI of the cervical spine confirmed degeneration at the C5-C6 and C6-C7 levels, but without cervical radiculopathy. A lumbar puncture revealed no signs of inflammation, and serology testing for Lyme disease came back negative. Serum inflammatory markers remained within normal ranges, and the search for various autoantibodies, including antiganglioside antibodies, also yielded negative results. An electromyogram (EMG) was performed, revealing severe axonal denervation of the suprascapular nerve that did not respond to stimulation (**Figure 1**). The clinical presentation and EMG data led to the diagnosis of Parsonage-Turner syndrome. Given the temporal relationship with the oncological treatment and the absence of alternative triggers such as infection, vaccination, or mechanical events, the causality of the sequential use of ICI-targeted therapy was established. However, the E/B regimen was continued in the absence of an alternative treatment for her metastatic melanoma. The patient recovered and regained function in her arm through physical rehabilitation. No specific treatment was introduced, and corticoids were not administered because the diagnosis was made at a stage where the muscles were already atrophied, and the goal was to prevent the development of additional steroid-induced myopathy.

Regular clinical evaluations and EMG assessments were conducted. At present, the patient is still undergoing treatment with E/B, with her oncological disease remaining stable. She is in good clinical condition, and there has been complete recovery of the suprascapular nerve.

## Discussion

Parsonage-Turner syndrome, also known as neuralgic amyotrophy, is a rare condition that primarily affects males. It was first described in 1948 by Parsonage and Turner (3). The evolution of neuralgic paresthesia typically consists of three phases: 1) a painful phase characterized by severe acute pain in the upper arm and shoulder, 2) the development of muscular weakness a few days after the onset of pain, and 3) muscle atrophy that can be observed a few weeks later. While the exact cause of Parsonage-Turner syndrome remains largely unknown, some triggers have been identified, including infectious episodes, vaccination, surgery, connective tissue disorders, or pregnancy (4). The choice of treatment depends on the stage of the syndrome. In the initial stage, the primary treatments involve pain relief medications, and corticosteroids have also shown some effectiveness. Ultimately, the recovery largely depends on rehabilitation, which can be initiated immediately after the painful phase. It’s worth noting that some cases of neuralgic amyotrophy have also been reported following antibiotic treatments (5). Combination therapy with BRAF/MEK inhibitors has significantly improved the prognosis of patients with BRAF-mutant metastatic melanoma. In the COLOMBUS study, patients receiving encorafenib plus binimetinib achieved a five-year overall survival (OS) rate of 34.7% (95% CI: 28.0-41.5%), in contrast to 21.4% (95% CI: 15.7-27.8%) for vemurafenib and 34.9% (95% CI: 27.9-42%) for encorafenib. The five-year progression-free survival (PFS) was also superior for encorafenib plus binimetinib, with a rate of 22.9% and a median duration of response of 18.6 months. In comparison, the five-year PFS rate for encorafenib and vemurafenib was 19.3% and 10.2%, respectively, with median durations of response of 15.5 and 12.3 months (6).

Only a few neurological adverse events related to the use of BRAF/MEK inhibitors have been documented, primarily in the form of case reports (7–9). A recent pharmacological study reported 15 cases of peripheral neuropathy with two primary phenotypes: a symmetric, axonal, length-dependent polyneuropathy, and a demyelinating polyradiculoneuropathy (10). Additional neurological adverse events that have been reported include myasthenia, reversible posterior leukoencephalitis, and facial diplegia (11–13). Meirson et al. observed that the combination of E/B was associated with an increased risk of peripheral neuropathy, including Guillain-Barré syndrome (14).

Our case report underscores the potential causality of the prior ICI regimen, particularly the use of ICI combinations. Indeed, ICIs can lead to various neurological immune-related adverse events (irAEs), accounting for approximately 1% of all irAEs. These neurological irAEs encompass conditions such as meningitis, peripheral neuropathy, and encephalitis. Two cases of Parsonage-Turner syndrome have been described in relation to ICIs. In one case, it was induced by pembrolizumab, with symptoms appearing rapidly, just two weeks after the initiation of ICIs (15, 16). In the second case, which was induced by nivolumab, symptoms manifested later, approximately three months after the initial ICI infusion, and the patient recovered upon discontinuation of ICIs. However, this patient displayed atypical elements for a Parsonage-Turner syndrome, such as bilateral involvement and a sensitive component (17). Additionally, Alhammad et al. reported two cases of brachial plexus neuritis associated with ICIs (**Table 1**) (18). The authors proposed a different pathophysiologic immune mechanism compared to the typical Parsonage-Turner syndrome, given the presence of atypical features: rapid onset of symptoms, trunk involvement, response to corticosteroids, and the absence of amyotrophy.

**Table 1:** Characteristics and outcomes of patients who underwent a Parsonage-Turner syndrome.

Case	Age/gender	Primary tumor/stage	Regimen used	Onset (weeks after start of treatment)	Treatment of side effect	Outcome of side effect
Our case	42yo/F	Metastatic melanoma	Anti BRAK anti MEK	1	Rehabilitation	Resolved
Keerty et al. 2021 (15)	55yo/M	Metastatic lung cancer	pembrolizumab	2	Prednisone for 2 weeks at 60mg	Permanant changes (persistance of muscles atrophy)
Porambo and al. 2019 (17)	71yo/M	Metastatic lung cancer	nivolumab	12	nivolumab discontinuation	Resolved
Alhammad and al. 2017 (18)	56/M	Metastatic melanoma	pembrolizumab	27	High-dose intravenous methylprednisolone then Oral prednisone at 1 mg/kg per day	Resolved
	50yo/F	Metastatic renal cell carcinoma	nivolumab	27	High-dose intravenous methylprednisolone then Oral prednisone at 1 mg/kg per day	Resolved

While we cannot definitively rule out the involvement of ICIs, as it has been described in the literature, the rapid onset of symptoms immediately after the initiation of combination therapy leans in favor of its potential causality. Parsonage-Turner syndrome typically occurs within a few days following triggering factors.

It’s worth noting that the treatment of neurological irAEs generally necessitates the use of glucocorticoids, and patients who do not respond to this first-line treatment may require additional immunosuppressive or immunomodulatory therapies. In our patient, improvement without the use of immunosuppressive drugs may lend support to the potential role of targeted therapy. However, we cannot draw a firm conclusion since it could be attributed to the natural history of nerve regeneration or the kinetics of ICIs following several weeks of discontinuation.

To the best of our knowledge, we are reporting the first case of Parsonage-Turner syndrome in a melanoma patient treated with ICIs followed by targeted therapy. Targeted therapy and a sequential strategy of ICI and targeted therapy are likely to play a crucial role in the treatment of advanced melanoma, as well as in the adjuvant setting, including stages IIb and IIc (COLOMBUS-AD study). As a result, our case highlights a new potential adverse event associated with these therapies, providing valuable information for clinicians managing such patients.

## Data availability statement

The original contributions presented in the study are included in the article/supplementary material. Further inquiries can be directed to the corresponding author.

## Ethics statement

Written informed consent was obtained from the individual(s) for the publication of any potentially identifiable images or data included in this article.

## Author contributions

CB: Writing – original draft. FD: Writing – review & editing. MF-R: Writing – review & editing, Investigation. MK: Investigation, Writing – review & editing. EG: Writing – review & editing, Supervision.

## References

[1] Dummer R Ascierto PA Gogas HJ Arance A Mandala M Liszkay G . Overall survival in patients with BRAF-mutant melanoma receiving encorafenib plus binimetinib versus vemurafenib or encorafenib (COLUMBUS): a multicentre, open-label, randomised, phase 3 trial. Lancet Oncol (2018) 19(10):1315–27. doi: 10.1016/S1470-2045(18)30497-2 30219628

[2] Long GV Hauschild A Santinami M Atkinson V Mandalà M Chiarion-Sileni V . Adjuvant dabrafenib plus trametinib in stage III BRAF-mutated melanoma. N Engl J Med (2017) 377(19):1813–23. doi: 10.1056/NEJMoa1708539 28891408

[3] Parsonage MJ Turner JWA . Neuralgic amyotrophy the shoulder-girdle syndrome. Lancet (1948) 251(6513):973–8. doi: 10.1016/S0140-6736(48)90611-4 18866299

[4] Feinberg JH Radecki J . Parsonage-turner syndrome. HSS J Musculoskelet J Hosp Spec Surg (2010) 6(2):199–205. doi: 10.1007/s11420-010-9176-x PMC2926354 21886536

[5] Finstad K Guajardo JR Scoville C . Neuralgic amyotrophy associated with antibiotic therapy. Ann Pharmacother (2008) 42(9):1344–7. doi: 10.1345/aph.1L185 18682542

[6] Dummer R Flaherty K Robert C Arance AM de Groot JW Garbe C . Five-year overall survival (OS) in COLUMBUS: A randomized phase 3 trial of encorafenib plus binimetinib versus vemurafenib or encorafenib in patients (pts) with BRAF V600-mutant melanoma. J Clin Oncol (2021) 39(15_suppl):9507–7. doi: 10.1200/JCO.2021.39.15_suppl.9507 PMC9916040 35862871

[7] Maurice C Marcus B Mason W . Guillain-barre syndrome after treatment with dabrafenib for metastatic recurrent melanoma. (P4.232). Neurology (2015) 84(14 Supplement).

[8] Batra J Ankireddypalli A Kanugula AK Gorle S Kaur J . Guillain-barre syndrome secondary to the use of dabrafenib and trametinib for the treatment of advanced thyroid carcinoma. Cureus (2023) 15(2):e35069. doi: 10.7759/cureus.35069 36819948 PMC9937682

[9] Velter C Libenciuc C Routier E Mateus C Fahmy J Ghoufi L . Neurotoxicity induced by targeted therapies in patients treated for metastatic melanoma. Eur J Cancer Oxf Engl (2019) 111:8–11. doi: 10.1016/j.ejca.2019.01.017 30798086

[10] Picca A Birzu C Berzero G Sanchez-Pena P Gaboriau L Vidil F . Peripheral neuropathies after BRAF and/or MEK inhibitor treatment: A pharmacovigilance study. Br J Clin Pharmacol (2022) 88(11):4941–9. doi: 10.1111/bcp.15513 36028463

[11] Zaloum A Falet JPR Elkrief A Chalk C . Myasthenia gravis following dabrafenib and trametinib for metastatic melanoma. Neurology (2020) 94(7):322–3. doi: 10.1212/WNL.0000000000008860 31888971

[12] Shailesh FNU Singh M Tiwari U Hutchins LF . Vemurafenib-induced bilateral facial palsy. J Postgrad Med (2014) 60(2):187–8. doi: 10.4103/0022-3859.132339 24823520

[13] Stefanou MI Gepfner-Tuma I Brendle C Kowarik M Meiwes A Eigentler T . Posterior reversible encephalopathy syndrome in a melanoma patient with dabrafenib and trametinib treatment following immunotherapy. JDDG J Dtsch Dermatol Ges (2020) 18(2):136–9. doi: 10.1111/ddg.13991 31814289

[14] Meirson T Asher N Bomze D Markel G . Safety of BRAF+MEK inhibitor combinations: severe adverse event evaluation. Cancers (2020) 12(6):1650. doi: 10.3390/cancers12061650 32580351 PMC7352287

[15] Keerty D Peguero E . Pembrolizumab-induced parsonage-turner syndrome. Neurol Clin Pract (2021) 11(5):e781–783. doi: 10.1212/CPJ.0000000000000994 PMC8610514 34840909

[16] Johnson DB Manouchehri A Haugh AM Quach HT Balko JM Lebrun-Vignes B . Neurologic toxicity associated with immune checkpoint inhibitors: a pharmacovigilance study. J Immunother Cancer (2019) 7(1):134. doi: 10.1186/s40425-019-0617-x 31118078 PMC6530194

[17] Porambo ME Sedarsky KE Elliott EJ Theeler BJ Smith JK . Nivolumab-induced neuralgic amyotrophy with hourglass-like constriction of the anterior interosseous nerve. Muscle Nerve (2019) 59(6):E40–2. doi: 10.1002/mus.26454 30809822

[18] Alhammad RM Dronca RS KottsChade LA . Brachial plexus neuritis associated with anti-programmed cell death-1 antibodies: report of 2 cases. Mayo Clin Proc Innov Qual Outcomes (2017) 1(2):192–7. doi: 10.1016/j.mayocpiqo.2017.07.004 PMC6134904 30225416

